# Integrating pharmacists into aged care facilities to improve the quality use of medicine (PiRACF Study): protocol for a cluster randomised controlled trial

**DOI:** 10.1186/s13063-021-05335-0

**Published:** 2021-06-11

**Authors:** Sam Kosari, Jane Koerner, Mark Naunton, Gregory M. Peterson, Ibrahim Haider, Emily Lancsar, David Wright, Theo Niyonsenga, Rachel Davey

**Affiliations:** 1 Discipline of Pharmacy, Faculty of Health, University of Canberra, Bruce, ACT Australia; 2 Health Research Institute, Faculty of Health, University of Canberra, Bruce, ACT Australia; 3 School of Pharmacy and Pharmacology, University of Tasmania, Hobart, TAS Australia; 4 Department of Health Services Research and Policy, Research School of Population Health, Australian National University, Acton, ACT Australia; 5 School of Pharmacy, University of East Anglia, Norwich, UK

**Keywords:** Residential aged care facility, Aged care, Care home, Quality use of medicines, Elderly, Potentially inappropriate medicine, Pharmacists, Cluster randomised controlled trial

## Abstract

**Background:**

Medication management in residential aged care facilities is an ongoing concern. Numerous studies have reported high rates of inappropriate prescribing and medication use in aged care facilities, which contribute to residents’ adverse health outcomes. There is a need for new models of care that enhance inter-disciplinary collaboration between residential aged care facility staff and healthcare professionals, to improve medication management. Pilot research has demonstrated the feasibility and benefits of integrating a pharmacist into the aged care facility team to improve the quality use of medicines. This protocol describes the design and methods for a cluster randomised controlled trial to evaluate the outcomes and conduct economic evaluation of a service model where on-site pharmacists are integrated into residential aged care facility healthcare teams to improve medication management.

**Methods:**

Intervention aged care facilities will employ on-site pharmacists to work as part of their healthcare teams 2 to 2.5 days per week for 12 months. On-site pharmacists, in collaboration with facility nurses, prescribers, community pharmacists, residents and families will conduct medication management activities to improve the quality use of medicines. Aged care facilities in the control group will continue usual care. The target sample size is 1188 residents from a minimum of 13 aged care facilities. The primary outcome is the appropriateness of prescribing, measured by the proportion of residents who are prescribed at least one potentially inappropriate medicine according to the 2019 Beers Criteria. Secondary outcomes include hospital and emergency department presentations, fall rates, prevalence and dose of antipsychotics and benzodiazepines, Anticholinergic Cognitive Burden Score, staff influenza vaccination rate, time spent on medication rounds, appropriateness of dose form modification and completeness of resident’s allergy and adverse drug reaction documentation. A cost-consequence and cost-effectiveness analysis will be embedded in the trial.

**Discussion:**

The results of this study will provide information on clinical and economic outcomes of a model that integrates on-site pharmacists into Australian residential aged care facilities. The results will provide policymakers with recommendations relevant to further implementation of this model.

**Trial registration:**

ACTRN12620000430932. Registered on 1 April 2020 with ANZCTR

## Background

Older adults residing in residential aged care facilities (RACFs) generally have complex co-morbidities and are prescribed a large number of different medications [1]. Studies have reported that, on average, RACF residents take between 9 and 11 regular medications [2–4]. Polypharmacy increases the risk of medication-related problems and adverse drug events, including hospitalisations, placing a significant burden on residents and economic cost on the health care system [5–7]. Australian studies have shown that almost all RACF residents have at least one medication-related problem [4, 8–11] and between 30% and 73% of residents are prescribed at least one potentially inappropriate medication (PIM) [4, 12–18]. According to a recent meta-analysis of 33 international studies, the use of PIM is significantly associated with an increased risk of hospitalisation in the older population, and the risk was higher in those who took more than one PIM [19]. Additionally, PIMs are associated with other potential adverse outcomes in older indivisuals, including fall, fracture, cognitive decline, delirium, stroke and cardiovascular events [20, 21].

Among PIMs, sedatives, antipsychotics and drugs with anticholinergic properties are particularly associated with greater risk of harm. A large Australian cohort study among 11,368 residents found that 61% were taking psychotropic medications, with the majority of these agents having sedative properties that can contribute to falls or confusion [22]. The over-use of psychotropic medications has been recently highlighted in the interim report of the Australian Royal Commission into Aged Care Quality and Safety [23]. Australian studies have reported that over 20% of RACF residents were taking antipsychotics regularly [22, 24], and the duration of antipsychotic use was longer than recommended [25–27]. Prolonged use of antipsychotics in older people is linked with increased risk of hospitalisation, hip fracture, peneumonia, stroke and death [28, 29]. Another large Australian study [30] of 17,000 RACF residents reported that 46% were taking drugs with moderate to strong anticholinergic effects; these drugs can contribute to cognitive and functional decline, delirium, worsening dementia, and increased mortality in older people [31].

Additionally, over-prescribing, using medicines longer than recommended, and drug interactions affect medication safety in aged care residents. The Australian 2018 Aged Care National Antimicrobial Prescribing Survey reported that 10% of residents were taking an antibiotic on the day of the survey, and about two thirds of these prescriptions were lacking relevant documentation of sign and symptoms to justify the need for antibiotic use [32]. Another large Australian study reported that more than 50% of residents were prescribed proton pump inhibitors with a median duration of use of 360 days in the year, while the recommended duration of use is 8 weeks [27]. Over-prescribing can also lead to unwanted drug interactions; a retrospective study of aged care resident’s medication records showed that 16% of residents were at high risk of drug-induced QT prolongation and potential arrhythmia due to polypharmacy [33]. Overall, many published studies highlight the need to improve medication management in RACFs. It is an area where pharmacists, doctors and nurses can work together, ensuring improved medication safety and quality use of medicines for residents [34].

Amongst the factors affecting medication safety and quality use of medicines in RACFs, lack of accessibility to pharmacists and doctors, and poor interdisciplinary collaboration were highlighted in a recent systematic review of international studies [35]. Consistent with these findings, the Australian Medical Association highlighted the “extremely urgent” need to increase the number of health care professionals in RACFs [36]. General practitioners (GPs), nurses and pharmacists are the key health professionals involved in the prescribing, administration and supply of medicines. Since these health professionals are generally not co-located, there are significant limitations in access, communication [37] and coordination of medication management processes [1] for aged care residents.

In Australia, there are two government-funded pharmacist-led services in place that aim to improve medication management in RACFs: (i) residential medication management review (RMMR) program [38] and (ii) quality use of medicine (QUM) service [39]. The RMMR for RACF residents has been in place since 1997 [37] and is similar to “clinical medication reviews” in the UK, “comprehensive medication reviews” in the USA and “MedsCheck LTC” in Canada [40–42]. The RMMR program enables GPs to refer RACF residents to accredited pharmacists to receive a medication review every 24 months or when there is a clinical need [43]. Although the RMMR service has been shown to be an effective strategy to identify and resolve medication-related problems and improve quality use of medicines for RACF residents [2], the service has logistical limitations. These include physical separation of community pharmacies, RMMR pharmacists and RACFs which leads to lack of timely access to pharmacist services when residents need them most [37]. Additionally, access to clinical pharmacists to conduct RMMRs for RACF residents is limited to periodic visits to the facility. Consequently, pharmacists performing RMMRs may not have a thorough understanding of the resident and may not be familiar with the facility staff and organisational structure, resulting in limited effectiveness of their activities within RACFs [44]. Other limitations of RMMR include limited involvement of pharmacists in the implementation and follow-up of recommendations and inconsistency in the level of collaboration between the health professionals in the RMMR processes [45]. QUM services are funded by the Commonwealth Department of Health for pharmacists to visit RACFS and conduct education to improve practices and procedures related to medication use. QUM services are intended to improve the medication management at the RACF level (e.g. through audits and staff education) [39, 46]; however, there has been little research to explore the effectiveness of this service [37].

Integrating an on-site pharmacist as part of the RACF health care team may address the gap in provision of medication management practices, policies and processes. On-site pharmacists, in collaboration with nurses, GPs, specialists, community pharmacists, residents and families will conduct medication management activities to improve the quality use of medicines at the facility [47–50]. This new model can improve communication among the healthcare team and enhance resident and family’s involvement in medication management decisions for individuals [48], leading to improved person-centred care. At the facility level, the on-site pharmacist can develop and enhance RACF policies and procedures for overall medication management [44]. These system improvement activities include reviewing and enhancing medication ordering, storage and administration processes, as well as conducting staff education, providing medication information, responding to medication utilisation reports, developing clinical referral pathways and contributing to staff and resident influenza vaccination.

A proposed model of integrated on-site pharmacist services into the RACF health care team was examined in a pilot study which was conducted by the lead author [47–51]. The conceptual foundation of the new model was to improve multi-disciplinary care, communication and collaboration in RACF’s healthcare team to enhance medication management [47, 48]. The findings of the pilot study indicated that the integration of a pharmacist into a RACF was feasible and acceptable to RACF staff, residents and GPs and resulted in improved medication administration and clinical documentation [47], increased provision of education for nursing and carer staff to promote the quality use of medicines and prevent medication administration errors [48], and enhanced staff influenza vaccination rates [49]. The positive findings of the pilot study informed the allocation of program funds from the Australian Department of Health to implement and evaluate this model in RACFs in the Australian Capital Territory (ACT).

The aim of this larger study is therefore to conduct a cluster randomised controlled trial (RCT) to evaluate if integrating pharmacists into RACFs, improves medication management in RACFs in the ACT, Australia. Objectives of the study include determining if this new integrated model (i) improves appropriateness of prescribing for RACF residents, as determined by the use of PIMs according to 2019 Beers Criteria [52], (ii) reduces RACF residents’ Emergency Department (ED) presentations and hospital admissions, (iii) improves other quality use of medicine indicators at the resident and facility levels, and (iv) is cost-effective.

## Methods

### Study design

This is a cluster RCT in RACFs in the ACT, Australia, with RACFs as the unit of randomisation. Participating RACFs will be randomised into either an intervention or control group. RACFs in the intervention group, in addition to ‘usual care’, will each employ an on-site pharmacist as member of the healthcare team. RACFs in the control group will continue ‘usual care’ that includes receiving government funded RMMR and QUM services from visiting pharmacists. Intervention and control groups will be recruited and randomised in staggered groups which will run in parallel.

### Participants

#### RACFs

All RACFs in the ACT that are nationally accredited facilities will be invited to participate in the trial. RACFs that have less than 20 beds will be excluded. There are a total number of 1978 RACF beds in facilities in the ACT, and the ACT had a population of 431,000 in 2020 [53].

#### Residents

Permanent residents of included RACFs will be included in the study unless they specifically request their data not to be included in the trial. Respite (non-permanent) residents will be excluded.

#### Pharmacists

Qualified pharmacists will be recruited through open expressions of interest sent to pharmacy professional groups and associations. The selection criteria for pharmacists include having registration with the Australian Health Practitioner Regulation Agency, accreditation to conduct medication reviews by the Australian Association of Consultant Pharmacy or equivalent hospital or geriatric clinical pharmacy experience, and accreditation to conduct vaccination. A list of eligible pharmacists will be provided to the intervention RACFs, who will employ pharmacists as per their organisational policy. Salaries for pharmacists will be funded by the research grant; however, they will be directly employed by RACFs as RACF staff members.

### Recruitment process

**Fig. 1:**
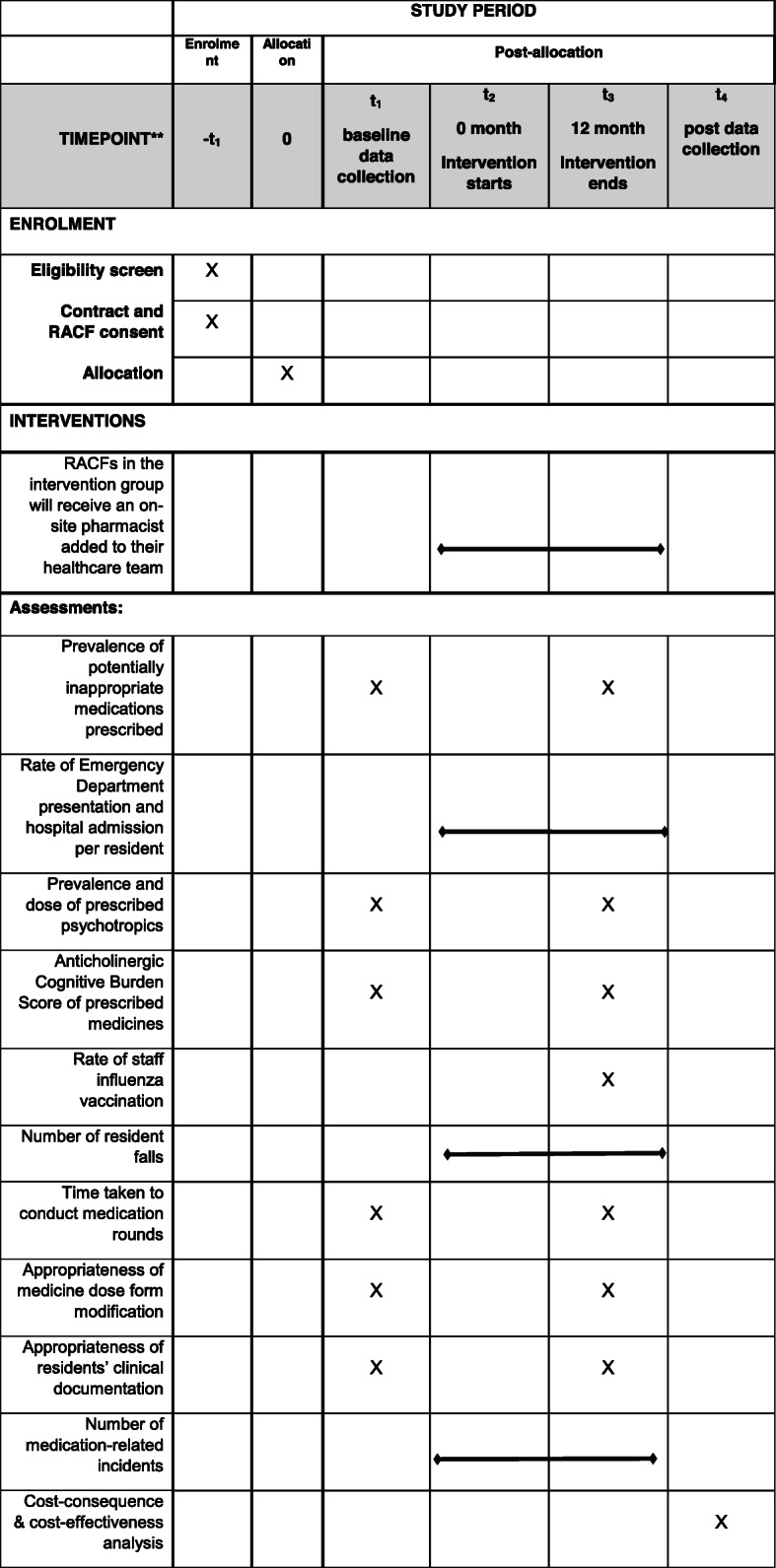
Study timeline

All RACFs in the ACT, Australia, that meet the inclusion criteria will be invited to participate in this study. After being provided information on the nature of the study and data required, each RACF will agree to participate through a signed contract. Recruitment will be staggered over a period of 6 months or until the sample size achieved. The recruitment and study timelines are shown in Fig. 1.

### Randomisation and blinding

Randomisation will be at the facility level. RACFs will be randomised into either intervention or control group through computer-generated allocation by an independent researcher external to the research team. Randomisation will be stratified by size of facility. Due to the nature of the intervention, the trial participants will not be blinded.

### Intervention and model of care

RACFs in the intervention arm will have a pharmacist employed by their organisation as part of their health care team for 2 to 2.5 days a week for 12 months. Intervention pharmacists can work in up to 2 RACFs. Pharmacists will report to RACF managers. They will conduct resident and facility level activities that are within their current scope of practice as a health professional registered with Australian Health Professional Registered Agency.

The intervention (model of care) was informed by the findings from the pilot study [47–51] and discussion with RACF managers, GPs, pharmacists and a consumer representative who participated in the pilot. Components of this model of care are informed by integration of pharmacists into non-dispensing primacy care roles [37, 54]. Components and how they differ from usual care are presented in Table 1.

**Table 1:** Key components and comparison between existing and proposed model

Key component	Existing model	Proposed model
Governance and service structure	RMMR & QUM activities are conducted by independent pharmacist (who are contractors) on visitational basis.	Pharmacist is employed by the RACF and is incorporated into RACFs care team. Pharmacist works within RACFs clinical governance structures.
Multi-disciplinary care (including resident and family)	Pharmacist is not incorporated into the RACF care team. They visit RACF at semi-regular intervals, provide medication advice to GPs through RMMRs and provide quality improvement projects.	Pharmacist is incorporated into the RACF care team and has contact with residents, families, GPs and prescribers, nurses and care staff. The pharmacist is available on-site at RACFs and involves residents and families into decision-making processes to improve medication management.
Reciprocal interdependence	Pharmacist provides medication review as an add-on service to assist GPs with quality of prescribing. However, they are not incorporated into the RACF care teams.	Multi-disciplinary team members, including pharmacists, nurses, carers, GPs and prescribers, community pharmacists, residents and families engage in shared decision making and work together to achieve goals.
Communication	Pharmacist communicates medication-related issues about individual residents to the GPs, usually through RMMR. GPs communicate medication changes to RACF nurses.	Pharmacist communicates and coordinates medication-related issues directly with GPs, nurses, carers, residents, community pharmacy and hospital.
Collaboration	Pharmacist usually collaborates with GPs to conduct RMMR.	Pharmacist closely collaborates on a regular basis with nurses, aged care staff and management, GPs and other prescribers, visiting pharmacists, community pharmacy, residents, families and hospital.
Sharing and access to information	Pharmacist has limited access to residents’ clinical records, which may include laboratory reports, while GPs and nurses have full access to clinical records.	All team members, including the pharmacist, will have full access to residents’ records, current medication lists, information about allergies, lab results, notes, procedures, and hospital discharge summaries.
Coordinated care/outcomes	Pharmacist provides once-off advice and opinion to GPs in RMMRs (including 2 follow-ups) but are not involved in implementing medication management changes or ongoing monitoring.	Residents’ treatment goals and outcomes are coordinated within the team of nurses, carers, pharmacist, GPs and other service providers. Pharmacist is involved in providing advice to GPs, prescribers and the RACF care team, and in implementing residents care plans and goals of care. Pharmacist also contributes to improving RACF medication management policies and procedures.

Pharmacist activities in intervention RACFs include the following:

- Performing medication reviews in collaboration with residents, families, prescribers and nurses
- Identifying residents at high risk of medication-related harm and hospitalisation, and prioritising interventions to address them
- Medication reconciliation and review at transition of care
- Participating in case conferences with GPs, palliative care team, families and residents
- Reviewing and optimising medication administration rounds
- Updating and improving resident records including clinical and care information
- Answering medication-related queries from residents, families and staff
- Conducting regular clinical audits to identify medication-related problems
- Educating residents, families and RACF staff about medication-related issues
- Improving the RACF’s medication management policies and procedures
- Participating in relevant RACF committees and meetings including Medication Advisory Committee, Quality and Safety meetings, Falls Review Committee, and Medication Incidents Review Committee
- Improving influenza vaccination rates of staff and residents

Pharmacists in intervention sites will not be permitted to conduct RMMR or QUM services. RACFs will receive these services from existing providers as a part of usual care.

### Pharmacist training and support

Pharmacists will participate in mandatory training before commencing in RACFs, including an initial full-day overview of clinical pharmacy practice in the aged care setting, followed by a session focused on the pharmacist’s role in RACFs and the trial design and processes. Pharmacists will be provided with clinical and geriatric pharmacy resources including content on deprescribing, psychotropics, pain management, principles of medication review in aged care, Beers Criteria [52] and wound management.

The study team will meet face to face with pharmacists monthly to discuss potential problems and address questions. Furthermore, pharmacists will be invited to participate in quarterly meetings held by the study team to discuss study activities. An online Microsoft Teams will link on-site pharmacists to each other to facilitate a community of learning to discuss issues they are experiencing.

### Outcomes

All outcome measures will be collected from both intervention and control RACFs and compared as below.

#### Primary outcome

- Change in proportion of residents who are prescribed at least one PIM (from baseline to 12 months) according to the 2019 Beers Criteria [52]

#### Secondary outcomes

- Rate of unplanned ED presentations and hospital admissions per resident collected from RACF records over 12 months
- Polypharmacy—number of regular medications
- Change in proportion of residents who are prescribed at least one psychotropic medicine (defined as antipsychotics and benzodiazepines), excluding those residents with major psychiatric diseases or epilepsy (from baseline to 12 months)
- Change in dose of psychotropic medicines (measured as chlorpromazine or diazepam equivalent daily dose [55] (from baseline to 12 months)
- Change in residents’ Anticholinergic Cognitive Burden Score (ACB) [56] (from baseline to 12 months)
- Rate of staff influenza vaccination measured at the end of influenza season, from RACF records
- Fall rate per resident, as documented from RACF fall records over 12 months
- Change in time spent on medication administration rounds per RACF, through observing randomly selected medication rounds [47] (from baseline to 12 months)
- Change in appropriateness of medicine dose form modification per RACF, through observing randomly selected medication rounds [47] (from baseline to 12 months)
- Change in proportion of residents who have drug allergies or adverse drug reactions documented in their RACF records (from baseline to 12 months)
- Number of medication-related incidents over 12 months
- Cost-consequence and cost-effectiveness of the intervention over 12 months

### Data collection

Data will be collected at RACF, resident and pharmacist levels throughout the 12-month trial period. RACFs’ characteristics (number of beds, number of permanent residents, resident profile and number of staff) will be collected through surveys with RACF managers. De-identified residents’ data for outcome measures will be collected by the research team visiting the facilities at baseline, each month and at 12 months. Randomly selected medication rounds will be observed to determine the time spent on medication rounds and assess the appropriateness of dose form modifications using the method described earlier [47]. In case of potential logistical limitations in light of COVID-19 and any future restriction of access to RACFs, RACF staff will collect the required data. On-site pharmacists in the intervention group will self-report their daily activities through an online diary using QUALTRICS. Details of data collection items and timing are listed in Table 2.

**Table 2:** Data collection details

Data	Data collection
**Facility level data**	
Number of permanent residents, proportion of residents with dementia, and proportion receiving the highest level of government funding	Baseline and at 12 months
Number of RACF registered nurses rostered during day/night/weekend	Baseline and at 12 months
Care staff turn-over reported by RACFs	Baseline
Total number of beds and bed occupancy rate	Baseline
Resident turn over	Monthly
Number of medication-related incidents	Monthly
Number of resident falls	Monthly
Time taken to conduct medication rounds	Baseline and at 12 months
% of staff/residents received influenza vaccination	At one time point
% of residents that have drug allergy and adverse drug reactions documented	Baseline and at 12 months
Number of GPs visiting residents in facility	Baseline
RACF managers perceived top 5 reasons for unplanned hospitalisations of residents in previous 12 months, and possible solutions for reducing these	Baseline
**Resident level data**	
Age and gender	Baseline
Date of admission and discharge and reason for discharge from the facility	Baseline and monthly
Diagnosis	Baseline and at 12 months
Number and list of regular and PRN medications including dosages	Baseline and at 12 months
Emergency Department visit/transfer*	Baseline and monthly
Hospital admissions* and length of hospital stay as determined by RACF residents’ records	Baseline and monthly
Reason for Emergency Department visit/admission to hospital - as determined by RACF residents’ records	Baseline and monthly
**Intervention pharmacist activity data**	
Daily activities and time taken to conduct each activity	Daily

*Outpatient appointments & scheduled procedures will not be included in hospital admission/emergency department visit data

### Sample size

It was estimated that a conventional RCT with randomisation of individuals would be able to detect a reduction from 60 to 40% of residents having at least one PIM [14], with a minimum of 106 residents in each arm (total of 212 residents in both arms) with a significance level of 5%, a power of 80% on equal allocation and a response rate of 85%. By adjusting for the loss of power due to clustering, with an intra-cluster correlation coefficient of 0.05 and a cluster size of 93 residents per RACF, the estimated sample size is (1 + [(93–1) x 0.05] =5.6 x 106) or 594 residents in each arm (1188 in both arms), equating to a minimum of 13 sites. The sample size was calculated using G*Power 3.1.9.4 [57].

The estimated prevalence of PIM in RACFs at baseline (60%) was based on previous studies in which the prevalence of PIM in Australian RACF residents were reported as 73% in 2018 [16], 49% in 2014 [14] and 56% in 2012 [58].

### Data management

Data will be collected from RACFs, by research staff. RACF staff will facilitate the collection of data from RACF digital and paper records. Collection of data will be onto a university laptop which is password-protected. Resident’s identifying details (e.g. names and date of birth) will be deleted prior to analysis. Residents will be given a unique study identifier to link data that will be stored in a secure place at the RACF. Data will be entered onto a central database developed with Microsoft Access and stored on the University of Canberra secure and password-protected data storage system. Access to the database will be by the key members of the research team with unique usernames and passwords. The servers are protected by firewalls and are maintained according to best practice. After the completion of the study, the database will remain on the university storage system for 5 years, as per National Health and Medical Research Council (NHMRC) guidelines.

### Statistical analysis

Descriptive statistics will be used to summarise and compare the data at the RACF level in each group at baseline and at the end of the trial, including primary and secondary outcomes as well as additional potential confounder variables (such as demographic profile, duration of residency, presence of dementia, Charlson comorbidity index [59] and number of medical conditions).

Bivariate analyses for group comparisons will use either t tests or ANOVA for data that are normally distributed, Mann-Whitney U and Kruskall-Wallis tests for data that are not normally distributed, or chi-square-based analysis for categorical outcome data. For within group comparisons, paired t tests and repeated measures ANOVAs will be used; if variables are not normally distributed, the Wilcoxon signed-ranks and Freedman tests will be used, while the McNemar test will be used for changes in proportions.

To determine the effect of the intervention on the outcome measures and the changes over time (between-within group effects), multilevel modelling methods, which take into consideration the hierarchical structure of the data (including clustering within RACF and repeated measurement occasions) will be applied. These modelling methods will include mixed-effects generalised linear models (Logistic and Poisson regression models) for binary and count outcomes as well as mixed-effects linear models for continuous outcomes assumed to have a normal distribution, or otherwise transformed to meet the assumption.

Analysis will be weighted by cluster size as required. Interactions and adjusting for demographic characteristics and other potential covariates will be included when deemed necessary. Residents who enter, die or move from RACFs after baseline data collection will have only one data point and will be included in the analysis. When data are missing at random, patterns of missing data will be evaluated, and potential predictors of missing responses will be investigated. Methodological attempts to fill in missing data will be extensively explored and applied as appropriate. These include single imputation approaches (such as regression imputation and nearest neighbours or hot-deck imputation) and multiple imputation approaches. Analysis will be conducted using either SPSS version 26 or STATA version 16. Significance level will be set at the usual 5% alpha-level (two-tailed where applicable). All estimated effects will be reported along with their 95% confidence intervals.

Since the sample size and power calculation have been devised based on the primary outcome and hypothesis, the level of adjustment and number of potential covariates to adjust for may be limited by the sample size and the response rate. Posterior power calculations will be performed based on available sample size and the secondary outcomes.

### Economic evaluation

A within trial cost consequence followed by a cost-effectiveness analysis will be conducted. The cost consequence analysis will explore the incremental impact (compared to the control arm) of the intervention on the disparate secondary outcomes, providing more information to decision makers in addition to a having a focus on the primary outcome. For the cost-effectiveness analysis, effectiveness will be measured in terms of the primary outcome—avoided PIM (reduction of the number of residents who take at least one PIM). A public health sector perspective will be used. All resource use will be valued in 2020/21 Australian dollars without discounting. Total costs for the intervention and control groups will be calculated, as well as average costs per participant, incorporating any additional costs relating to the delivery of the intervention (e.g. additional training, time that a GP spends on reviewing pharmacist recommendations). Resource use captured during the trial will include health service utilisation by each participant (ED visits, hospital admissions, ambulance transfer during the 12 months of control/intervention period; and medications used at baseline and at 12 months). Analogous multilevel modelling described above (controlling for differences in characteristics of participants and RACF clusters) will be used to estimate average cost per participant for both intervention and control groups. An incremental cost-effectiveness ratio (ICER) will be computed by comparing the costs and outcomes of the intervention and control groups. Results will be expressed as incremental cost per incremental reduction in the proportion of residents taking at least one PIM. Mean estimates will be used, and confidence values and sensitivity analysis will indicate the robustness and validity of the results and test any assumptions used. Uncertainty around the ICER will be explored using cost-effectiveness acceptability curves.

### Fidelity assessment

Fidelity in intervention sites will be assessed using Hasson’s Conceptual Framework [60], that assesses adherence against content, coverage, frequency and duration domains at the cluster level. First, 100% of pharmacist diaries in each intervention RACF will be assessed and cumulative number and proportion of activities will be calculated. Second, a random sample of 10% of resident’s medications reviews conducted by intervention pharmacists will be assessed by an experienced pharmacist to determine the appropriateness of medication reviews. Third, interviews with RACF managers, staff and pharmacists will further explore adherence to the trial activities. Intervention RACFs will be given a fidelity rating of high/medium/low based on the assessment.

### Trial management

The trial is overseen by the trial management group comprising chief investigators and the senior programme manager. The trial is advised by the governance committee organised by the funder, the ACT Primary Health Network (PHN) and comprises representative from RACFs, Pharmaceutical Society of Australian, Pharmacy Guild of Australia, Calvary hospital, a GP and a consumer representative. Potential protocol modifications by the trial management group will be communicated to the governance committee and human research ethics committees.

### Safety evaluation and reporting

RACFs are required to have clinical governance processes and complaints procedures in place. Criteria for monitoring the trial are informed by Stallard [61], whereby adverse events will be monitored and the trial ceased if there is evidence of harm. All adverse events will be entered into an *Adverse Event Log* and reported to external clinical consultants to determine whether or not they are considered causally related to study. For every adverse event, researchers and external consultants will provide an assessment of the severity, causal relationship to the study, outcomes and seriousness of the event, and document all actions and inform the Human Research Ethics Committee. In light of the COVID-19 pandemic, the research team will follow all RACF’s safety protocols and guidelines when they visit RACFs to ensure the safety of the residents and RACF staff.

## Discussion

The initial pilot study [47–51] confirmed the feasibility of the model, and no adverse events were identified. This is the first cluster RCT to our knowledge that investigates the effectiveness of integrating pharmacists in RACFs on improving medication management. The primary outcome is the appropriateness of prescribing that in a broader sense may represent an ideal for care [62]. Inappropriate prescribing has become an important public health concern worldwide [63] and is also prevalent in Australian RACFs [14, 16, 58]. In this trial, appropriateness of prescribing is measured using explicit Beers Criteria [52] which can be readily applied to a large sample of study participants with a high level of reliability and reproducibility [63]. Secondary outcomes include measures such as hospital admission and ED visit that are important from the public health, aged care industry and resident perspectives.

Medication management for older residents in RACFs is sub-optimal [4]. International evidence has demonstrated that pharmacist-led interventions in RACFs improve the quality use of medicines; however, the majority of these interventions were conducted by visiting pharmacists on once-off or limited visitation basis [34, 64]. There is a need for sustainable interventions to enable system level improvement in medication management practices in RACFs.

The study is using a staggered approach to the recruitment and intervention. Due to the impact of the recent COVID-19 pandemic on RACF’s workforce and operations, this staggering will provide the facilities with time to prepare and adapt to recent policy and procedural changes. These changes may impact on the study outcomes; for example, there may be changes in the number of regular healthcare staff in RACFs or residents may receive fewer GP and other visiting healthcare professional visits and this may impact the level of collaboration with pharmacists. Potential restrictions in visiting RACFs due to COVID-19 pandemic may affect the data collection processes. On-site pharmacists participating in this study will have accreditation to conduct medication review; however, they may be at different level of experience and skills, which may impact the quality of pharmacist activities in some RACFs. This will be further explored by assessing the fidelity of interventions that determines whether the intervention was conducted as planned across the intervention RACFs and includes an audit on the appropriateness of pharmacists’ medication reviews. Participating RACFs will be all within ACT which is a metropolitan area in Australia; thus, the findings may not be generalisable to RACFs located in rural and remote areas.

A number of limitations should be noted. PIMs are a proxy measure for appropriateness of prescribing, which represents an ideal level of care and is reliable in predicting adverse events [62]. The study does not include measurements of resident focused indicators such as Quality of Life, noting the difficulties in seeing changes in elderly frail population. The reporting of secondary outcomes is based on facility records, which may be under reported.

The study provides important information on clinical and economical outcomes of the model where on-site pharmacists are integrated into RACFs’ health care team to improve medication management. The results will provide policymakers with recommendations relevant to the potential further implementation of this model.

### Trial status

The study is being conducted according to the trial protocol version 3 revised on April 7, 2020. Recruitment began on Oct 28, 2019, and is anticipated to be completed by July 1, 2020.

## Data Availability

The trial dataset will not be made publicly available. Only investigators have access to the trial dataset.

## Abbreviations

RACF: Residential aged care facility
PIM: Potentially inappropriate medication
RMMR: Residential medication management review
GP: General practitioner
QUM: Quality use of medicines
RCT: Randomised controlled trial
ED: Emergency department
ICER: Incremental cost-effectiveness ratio
NHMRC: National Health and Medical Research Council
